# Leber Hereditary Optic Neuropathy and Epilepsy in a Mexican Patient

**DOI:** 10.7759/cureus.86663

**Published:** 2025-06-24

**Authors:** Emilio García Gómez, Daniel San-Juan, Lenin Sandoval Luna, Miguel Angel Morales Morales

**Affiliations:** 1 Epilepsy Clinic, Instituto Nacional de Neurología y Neurocirugía Manuel Velasco Suárez, Mexico City, MEX

**Keywords:** epilepsy, epilepsy genetics, leber hereditary optic neuropathy (lhon), neurology and epilepsy, neuro-opthalmology

## Abstract

This case describes a woman in her 30s who was diagnosed with Leber hereditary optic neuropathy (LHON), a genetic disorder causing vision loss associated with mitochondrial DNA mutations. Initially diagnosed in childhood, she also developed epilepsy in adolescence. Despite treatment with multiple anticonvulsants, her initial seizures were only partially controlled. Brain MRI and scalp EEG results indicated left temporal lobe epilepsy, and she ceased seizures after modification of her anticonvulsant medication regimen. This rare comorbidity highlights the complexity of LHON, which often presents with additional neurological symptoms beyond vision loss. It underscores the need for comprehensive treatment and further research into mitochondrial diseases and their broader neurological impact.

## Introduction

Genetic mutations associated with mitochondrial metabolism have been linked to neurological diseases such as epilepsy and multisystemic abnormalities [1]. Leber hereditary optic neuropathy (LHON) is a maternally transmitted disease caused by mutations in the mitochondrial genome, characterized by painless bilateral loss of central vision due to selective degeneration of retinal ganglion cells [2]. There are three mutation points in the mitochondrial DNA (mtDNA) involved in LHON: G17778A, T1448, and G3460A, which affect subunits 46 and 1 of the NADH dehydrogenase and are referred to as ND4, ND6, and ND1 mutations, respectively [3]. The typical age of disease onset is between 15 and 35 years. Males are more likely to be affected by LHON than females, with the reported ratio ranging from 4:1 to 5:1 [4]. In some cases, the ophthalmological signs may be complicated by additional neurological signs such as dystonia, parkinsonism, cerebellar ataxia, epilepsy, myoclonus, juvenile-onset encephalopathy, and psychiatric disturbances [5-8]. Few cases have been published about the presence of LHON and epilepsy with various phenotypes from different countries; however, this clinical coexistence has never been described in the Latino population. We present a case of a Mexican woman in her 30s with LHON and focal temporal epilepsy.

## Case presentation

**Figure 1:**
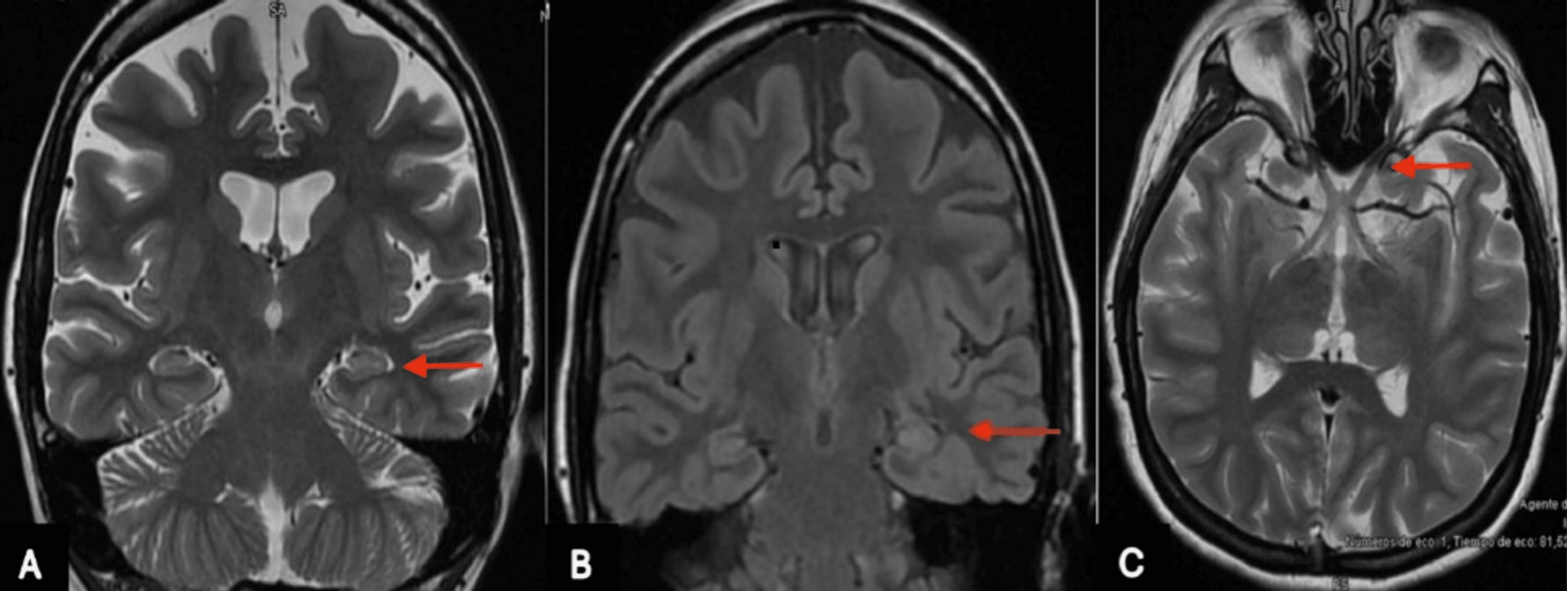
Coronal 3.0 T brain MRI (A: T2-weighted, B: FLAIR) shows a subtle increase in signal intensity in the left parahippocampal gyrus, more evident on the FLAIR sequence (red arrow), consistent with left mesial temporal sclerosis. Axial T2-weighted image (C) shows no abnormalities in the optic nerves (red arrow). FLAIR: fluid-attenuated inversion recovery

**Figure 2:**
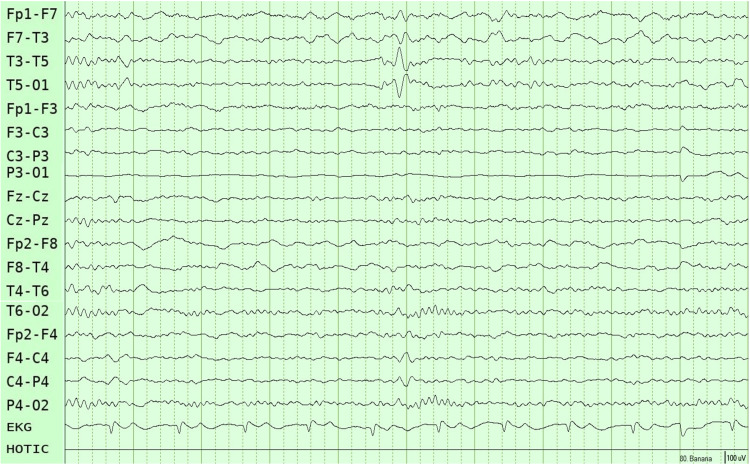
Ten-second sleep scalp EEG using a longitudinal bipolar montage (10–20 system), showing interictal epileptiform discharges over the left temporal region. Acquisition parameters: bandpass filter 0.3–70 Hz, notch filter 60 Hz, sensitivity 10 µV/mm.

A right-handed female patient in her 30s with a longstanding history of LHON presented for evaluation of her epilepsy. She experienced her first focal impaired awareness seizure at the age of 15 years, characterized by dizziness and transient loss of consciousness lasting approximately seconds to a minute, accompanied by postictal confusion, occurring at least once per month. However, she did not receive antiseizure medications or an epilepsy diagnosis during her initial medical evaluation, as her symptoms were incorrectly attributed to transient systemic hypotension episodes. The focal impaired awareness seizures were increasing in both frequency and intensity. At the age of 25, she experienced a focal to bilateral tonic-clonic seizure (FTBTC) lasting three minutes and followed by a post-ictal period characterized by drowsiness and fatigue for 10 minutes. She was promptly taken to a nearby hospital, where she underwent a brain MRI (Figure 1) and a scalp EEG (Figure 2), which revealed left mesial temporal sclerosis and left temporal epileptiform activity. 

She was treated with levetiracetam and valproate for three years; however, both types of seizures persisted. Additionally, she developed bilateral polycystic ovarian syndrome, which was managed with hormonal therapy due to symptoms of polymenorrhagia and abdominal pain. Although polycystic ovarian syndrome is a known adverse effect of valproate therapy, its occurrence may also be independently associated with epilepsy-particularly temporal lobe epilepsy-as suggested in previous studies [9].

The patient had no relevant family history of neurological or psychiatric disorders. Her perinatal history indicated an uneventful birth and normal neurodevelopmental milestones. There was also no history of febrile seizures or encephalitis. At 12 months of age, her parents noticed she did not fixate her gaze and blinked when exposed to intense light, progressively losing her vision until she was completely blind at the age of six years. Her electroretinogram and visual evoked potentials showed a lack of response, with no neurophysiological evidence of adequate light conduction of the visual pathway at the occipital cortex level. A genetic test detected a homoplasmic LHON 11778G>A mutation. These findings led to a diagnosis of LHON. No other genetic test was performed.

**Figure 3:**
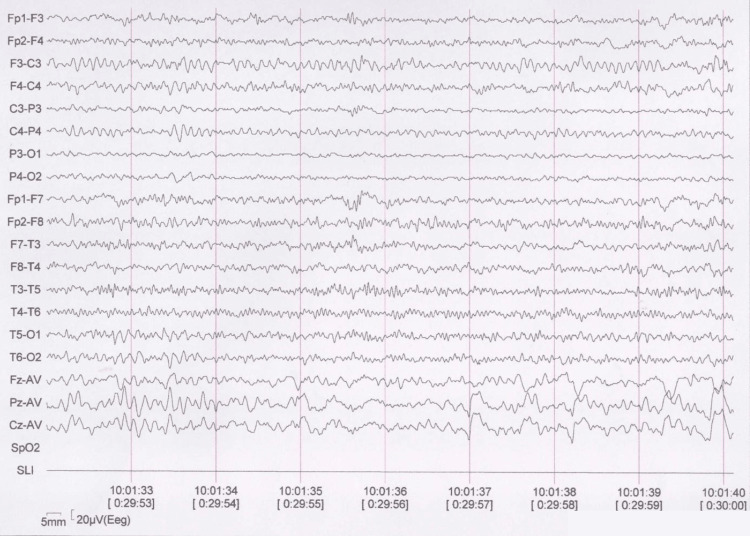
Awake scalp EEG using a longitudinal bipolar montage (10–20 system), showing normal background activity without epileptiform abnormalities. Recording duration: 30 minutes. Acquisition parameters: bandpass filter 1–70 Hz, notch filter 60 Hz, sensitivity 4 µV/mm.

Upon evaluation, physical examination revealed bilateral amaurosis and bilateral conductive hearing loss; otherwise, it was unremarkable. Given the initial refractory behavior of her focal epilepsy, her treatment regimen was modified to oral levetiracetam 1,500 mg BID, while valproate was discontinued, and lacosamide 300 mg/day was added. The patient has been monitored for a period of 12 months and remains seizure-free. Recent studies include a brain MRI, which continues to demonstrate minimal sclerosis of the left hippocampus, as well as a scalp EEG that does not exhibit any epileptiform activity (Figure 3).

## Discussion

This case presents a young female patient who became blind and was diagnosed with LHON during childhood and later developed focal temporal epilepsy in her teenage years. Petito et al. reported a case of earlier-onset LHON, accompanied by a history of neurological disorders like epilepsy and migraine [10]. Additionally, there are several other reports linking LHON with various neurological conditions, not only epilepsy but also cerebellar ataxia, dystonia, multiple sclerosis, parkinsonian syndrome, and supranuclear ophthalmoplegia [5,11,12]. Thus, LHON can be associated with other neurological conditions or clinical manifestations. However, it is also possible that these coexisting pathologies are independent and not related to each other. Given the variety of genetic alterations observed in LHON cases, it is not possible to establish a clear hypothesis regarding the relationship between specific LHON mutations and the development of particular associated conditions. These phenotypes have been termed “LHON plus syndromes” and have been linked to other mtDNA point mutations affecting oxidative phosphorylation complex I activity, differing from the three commonly seen in LHON [13]. However, unlike mitochondrial syndromes such as mitochondrial encephalomyopathy, lactic acidosis, and stroke-like episodes (MELAS), myoclonic epilepsy with ragged red fibers (MERRF), or Leigh syndrome-where epilepsy is a better-described feature-LHON has fewer reported associations with seizures. It is, therefore, possible that, in this case, mesial temporal sclerosis and epilepsy are not directly linked to the LHON diagnosis but instead represent an independent pathology occurring concurrently in the patient. From a genetic standpoint, mtDNA itself may contribute in various ways to LHON manifestation: the homo- or heteroplasmy conditions of the primary mutation, the presence of additional mtDNA mutations, and the number of mtDNA copies within cells [7].

The coexistence of LHON and epilepsy has been described in a few cases, none of which are in Latin America. Although there is currently no statistical data regarding the prevalence of LHON or LHON plus in Mexico, these conditions are likely misdiagnosed or underdiagnosed due to a lack of accessible diagnostic resources, such as genetic testing and imaging studies. These resources are not always available, and many patients may not have the financial means to obtain them. However, this was not the case with our patient.

The lack of information about LHON plus epilepsy does not allow us to choose the most effective antiseizure medication; nevertheless, according to similar cases, we can get rid of certain drugs that may make it worse.

In a female monozygotic twin with focal epilepsy, she developed manifestations of LHON after starting zonisamide monotherapy; then, treatment with levetiracetam and zonisamide was unsuccessful, and monotherapy with lamotrigine resulted in seizure freedom [7]. Another case involved a 43-year-old patient who suffered visual loss four years after initiating topiramate, initially diagnosed with bilateral toxic optic neuritis but later confirmed to carry the LHON 11778G>A mutation [14]. Topiramate was, therefore, discontinued, and levetiracetam was started to get seizure control. These two cases highlight the importance of avoiding certain drugs that might increase the probability of vision loss; topiramate and zonisamide are carbonic anhydrase inhibitors; therefore, both can trigger acute angle-closure glaucoma, eye movement disorders, diplopia, and blurred vision.

In two children with severe myoclonic epilepsy of infancy without SCN1A gene mutations, LHON onset included frequent myoclonic and tonic seizures, initially managed with valproate. Later, topiramate, levetiracetam, and lamotrigine were used without significant success [1]. Another case involved a 24-year-old Italian man with the m.3460G>A mutation who presented with rapid bilateral vision loss at age 16, followed by epilepsy a year later, managed with oxcarbazepine, topiramate, paroxetine, pregabalin, and lorazepam [15]. Two patients with temporal lobe epilepsy and LHON were reported, one managed with phenobarbital achieving adequate seizure control [16]. Another 12-year-old girl with the 11776G>A mtDNA mutation developed right focal temporal lobe epilepsy, refractory to treatment until a ketogenic diet achieved a moderate response [17]. Additionally, two patients with pharmacoresistant partial epilepsy displayed vision loss during pre-surgical assessments. One case involved a 26-year-old woman who experienced progressive vision loss following intracranial electrode implantation, with genetic analysis revealing the G11778A LHON mutation. The other one, a 31-year-old patient, reported progressive visual impairment during pre-surgical evaluation, leading to the diagnosis of a homoplasmic missense mutation at the nucleotide pair C4640A of the ND2 gene [18]. An overview of LHON with epilepsy is presented in Table 1.

**Table 1:** Overview of LHON cases associated with epilepsy. LHON: Leber hereditary optic neuropathy

Gene mutation	Epilepsy age of onset	Clinical features	Treatment	References
No reported	No reported	Bilateral temporal epilepsy/focal seizures	Lamotrigine	Petito et al. (2024) [10]
11778G>A	34 years	Temporal lobe epilepsy	Levetiracetam	Rinalduzzi et al. (2012) [14]
14484T>C; 15257G>A	6 months; 10 weeks	Myoclonic seizures; secondary generalized seizures	Topiramate, levetiracetam, and lamotrigine; lamotrigine monotherapy	Frye et al. (2011) [1]
m.3460G>A	13 years	Tonic-clonic seizures	Oxcarbazepine, topiramate, pregabalin, and lorazepam	Bianco et al. (2018) [15]
G3460A/ND1	7 years	Tonic-clonic seizures	Phenobarbital	Guerriero et al. (2011) [16]
11776G>A	14 months	Temporal lobe epilepsy	Ketogenic diet	Grazina et al. (2007) [17]
G11778A; C4640A/ND2	8 years; 21 years	Temporal lobe epilepsy; temporal lobe epilepsy	Surgical management	Niehusmann et al. (2011) [18]

Our patient experienced a similar clinical course, with bilateral vision loss due to LHON and subsequent temporal lobe epilepsy. Despite the complexity of these conditions, she achieved seizure freedom after trying three antiseizure medications. There is no history of febrile seizures or head trauma in any previous case report. However, a global development delay and family history of neurological disease and psychiatric disorders are presented in several cases of LHON plus. The underlying pathophysiological mechanisms linking LHON and epilepsy remain unclear but may involve mitochondrial dysfunction and oxidative stress pathways. Defective mitochondrial oxidative phosphorylation precipitates a bioenergetic crisis, leading to retinal ganglion cell failure. Reactive oxygen species are elevated due to altered electron flow through the mitochondrial respiratory chain caused by defective complex I, resulting in oxidative damage to DNA, proteins, and lipids [19]. The low production of ATP and reactive oxygen species can eventually cause cell death and visual loss in LHON patients [13,16]. Mitochondria are the primary source of cellular ATP and regulate Ca^2+^ homeostasis, implicating mitochondrial dysfunction in the pathophysiology of epilepsy [13]. Further research is warranted to elucidate the pathogenic links between these conditions and to optimize therapeutic strategies for affected individuals.

## Conclusions

This case highlights the rare co-occurrence of LHON and temporal lobe epilepsy in a Mexican female patient, emphasizing the potential multisystemic involvement of mitochondrial disorders. While the underlying mechanisms linking these two conditions remain to be fully elucidated, mitochondrial dysfunction and oxidative stress may play central roles. Nevertheless, it remains possible that LHON and temporal lobe epilepsy in this patient are two unrelated conditions that coincidentally coexist. The successful seizure control achieved through careful adjustment of antiseizure medication reinforces the importance of individualized therapeutic strategies, particularly avoiding drugs that may exacerbate mitochondrial-related visual impairment. This report underscores the need for greater clinical awareness, early diagnosis, and tailored management in patients with LHON and coexisting neurological manifestations.
